# Can a Black-Box AI Replace Costly DMA Testing?—A Case Study on Prediction and Optimization of Dynamic Mechanical Properties of 3D Printed Acrylonitrile Butadiene Styrene

**DOI:** 10.3390/ma15082855

**Published:** 2022-04-13

**Authors:** Ronak Vahed, Hamid R. Zareie Rajani, Abbas S. Milani

**Affiliations:** School of Engineering, University of British Columbia, Kelowna, BC V1V 1V7, Canada; ronak.vahed@gmail.com (R.V.); hamid.r.zareie@gmail.com (H.R.Z.R.)

**Keywords:** acrylonitrile butadiene styrene, additive manufacturing, artificial neural networks, dynamic mechanical properties, Particle Swarm Optimization

## Abstract

The complex and non-linear nature of material properties evolution during 3D printing continues to make experimental optimization of Fused Deposition Modeling (FDM) costly, thus entailing the development of mathematical predictive models. This paper proposes a two-stage methodology based on coupling *limited* data experiments with black-box AI modeling and then performing heuristic optimization, to enhance the viscoelastic properties of FDM processed acrylonitrile butadiene styrene (ABS). The effect of selected process parameters (including nozzle temperature, layer height, raster orientation and deposition speed) as well as their combinative effects are also studied. Specifically, in the first step, a Taguchi orthogonal array was employed to design the Dynamic Mechanical Analysis (DMA) experiments with a minimal number of runs, while considering different working conditions (temperatures) of the final prints. The significance of process parameters was measured using Lenth’s statistical method. Combinative effects of FDM parameters were noted to be highly nonlinear and complex. Next, artificial neural networks were trained to predict both the storage and loss moduli of the 3D printed samples, and consequently, the process parameters were optimized via Particle Swarm Optimization (PSO). The optimized process of the prints showed overall a closer behavior to that of the parent (unprocessed) ABS, when compared to the unoptimized set-up.

## 1. Introduction

**Figure 1:**
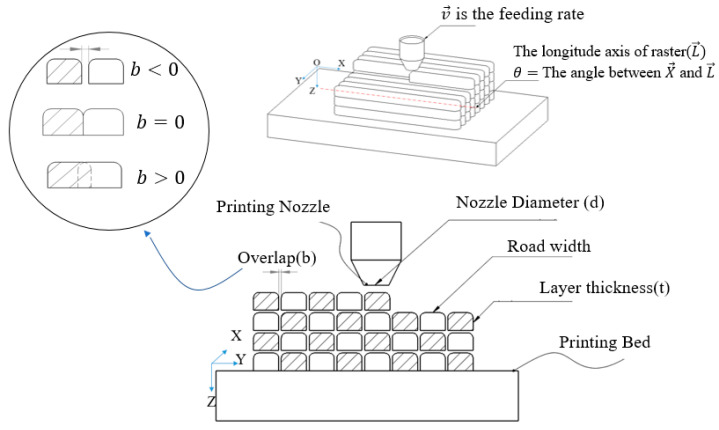
FDM main process parameters.

Fused Deposition Modeling (FDM) is increasingly being used as a reliable rapid prototyping tool in industries. However, an outstanding challenge in the field of additive manufacturing is yet to employ FDM to build high-quality end-use parts with minimal waste, while maintaining a high rate of production [1]. Exploiting the full potential of FDM for manufacturing requires the proper selection of process control factors through a good understanding of their nature [2]. Over years of evolving additive manufacturing techniques, several investigations have been performed to study the effect of FDM process parameters, e.g., [3,4,5]. In these studies, the road width (w), which is the width of the road deposited through the nozzle, layer thickness (t), which is the thickness of each 2D layer, feeding rate (v), which is the rate at which the thermoplastic filament is fed into the nozzle, nozzle temperature (NT), raster orientation (θ), which is the orientation of roads in each 2D layer, overlap (b), which is the amount of overlap between two adjacent roads, and nozzle diameter (d) have been introduced as the main effective process parameters [3,4,5]. Figure 1 schematically depicts the above-mentioned process parameters.

Assessment of FDM fabricated parts could be performed using several quality measures such as durability and static mechanical properties, dynamic mechanical properties, and manufacturing accuracy [6,7,8,9,10,11,12,13,14,15,16,17]. Despite the fact that thermoplastics are viscoelastic materials, due to the expensive and time-consuming nature of the dynamic mechanical analysis (DMA) tests, the time- and temperature-dependent mechanical properties of FDM processed parts have not been characterized as extensively as their static mechanical properties [3].

One of the earliest studies on dynamic mechanical properties of FDM processed parts goes back to the work of Chin Ang et al. [18], with respect to a few select process parameters including air gap, raster width, raster orientation, deposition profile, and layer height. In their study, the air gap and raster width were identified as the most effective process parameters to control the porosity and strength of processed parts. Furthermore, they claimed that there is a logarithmic relationship between mechanical properties and porosity, meaning that 3D printed scaffold parts with a lower porosity would show a higher strength. Later, Arivazhagan et al. [5] used the dynamic mechanical analysis (DMA) to examine the effects of road width, raster orientation, and nozzle temperature on viscosity and dynamic moduli of FDM processed Acrylonitrile Butadiene Styrene (ABS) samples. They showed that a raster orientation of 30°/60° and a road width of 0.454 mm improves the dynamic moduli of 3D-printed ABS. Mohamed et al. [19] considered layer thickness, overlap, raster angle, raster orientation, and road width as control factors to investigate the dynamic mechanical properties of FDM processed ABS. The results of their study indicated that the overlap and layer thickness are the most effective process parameters. Specifically, it was shown that a layer thickness of 0.3302 mm, a road width of 0.4572 mm, and an overlap of 0 mm with a raster angle of $0^{{}^{\circ}}$ can increase the dynamic moduli of ABS.

However, none of the above past studies on dynamic mechanical properties of FDM processed thermoplastics accounted for other important parameters including nozzle temperature and feeding rate. In addition, a lack of systematic experimental design (e.g., using the DOE methods) did not allow to fully account for the combined effects of various process parameters (most studies used a one-factor-at-a-time sensitivity analysis). This could lead to errors in analyzing the role of FDM parameters in the dynamic mechanical characteristics of 3D printing materials.

This paper presents a two-stage methodology to study the concurrent effects of multiple FDM process parameters including nozzle temperature, raster orientation, layer thickness, and feeding rate on dynamic mechanical properties of FDM processed ABS. Specifically, the variations of the dynamic moduli and glass transition temperature of ABS as a function of changes in four key FDM parameters (nozzle temperature, layer height, raster orientation and deposition speed) are analyzed using a Taguchi design of experiment (DOE). The latter not only minimizes the number of costly and time-consuming DMA tests, but also accounts for the combined effects of the FDM parameters. Once the required (limited) dataset was gathered through performing experiments, a series of artificial neural networks were developed and employed to predict the properties of the 3D prints, and consequently, the process parameters were optimized via a particle swarm optimization (PSO). Finally, the contribution and ranking of process parameters were identified.

## 2. Methods

### 2.1. Material and Fabrication

Owing to its relatively low melting temperature and high-quality surface finish, ABS is one of the most commonly used materials in 3D printing industries [20]. The initial ABS material in the current study was in the form of filaments, which were then fed into a 3D printer (MakerGear M2, Beachwood, OH, USA) to manufacture rectangular samples of 57 mm×14 mm×1.25 mm (length × width × thickness) for subsequent DMA characterization tests. The as-received filaments were extruded out of ABS POLYLAC^®^ by CHIMEI Corp. (Tainan City, Taiwan), with specifications shown in Table 1.

### 2.2. Dynamic Mechanical Analysis

Dynamic mechanical analysis (DMA) [21] characterizes the viscoelastic properties of materials, described by the storage modulus ($E^{\prime }$), loss modulus ($E^{\prime \prime }$), complex modulus ($E^{*}$), and tanδ [22]; tanδ is a dimensionless property, defined as the ratio of the loss modulus to the storage modulus. DMA also quantifies the glass transition temperature ($T_{g})$, which denotes the transition point between glassy and rubbery states [23]. In this study, the dynamic mechanical analyses of ABS 3D-printed samples were carried out using DMA Q800 (TA Instruments, New Castle, DE, USA). DMA testing was also conducted on original ABS filaments (length of 57 mm) prior to printing; this will be referred to as unprocessed material hereafter. The test specimens were prepared according to the DMA 800 manufacturer instructions.

### 2.3. Artificial Neural Network (ANN)

**Figure 2:**
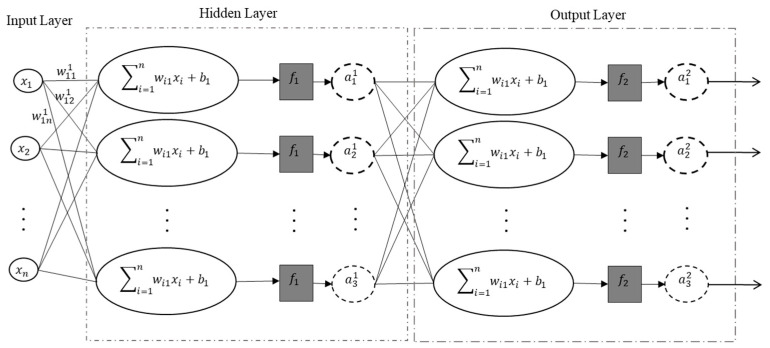
Architecture of artificial neural network with one hidden layer. The superscripts represent the layer number. The first and second subscripts, respectively, represent the input and neuron number that are associated together in each layer.

The Artificial Neural Network (ANN) modeling technique, inspired by the neurologic system of the brain, has received notable attention in recent years in the AI field. The ability to approximate complex non-linear relationships between input and output parameters in complex systems is the main advantage that has made the technique a useful predictive modeling tool in a wide range of applications [24]. In addition, ANNs are known to provide predictions with minimal prior assumptions, hence making them a useful black-box modeling means for unknown/complex systems, as opposed to more explicit, e.g., regression techniques that require a “pre-defined” form of input-output relationship. As depicted in Figure 2, an artificial neural network model is a collection of processing units called nodes (also called neurons). In a neural network, neurons are connected to each other by numerically assigned connections, known as weights $\left(W_{i}\right)$ and are fed by a signal from each input ($x_{i}$). The neuron’s scalar output (a) is the summation of the weighted inputs and the bias term (*b*) modified by a transfer function (f). Summation of weighted inputs is denoted by s and is presented as:

(1) $$s=w_{1}x_{1}+w_{2}x_{2}+\ldots +w_{n}x_{n}+b=\overset{n}{\underset{i=1}{\sum }}w_{i}x_{i}+b$$

The bias term acts similar to an input with the value of 1 and its existence in a network is not mandatory. However, it often improves the performance of the model [25,26]. It must be noted that weights and biases are adjustable terms that are updated through the learning algorithm. The transfer function is fixed throughout the whole process.

An ANN architecture consists of three layers: input, hidden, and output layers. The input layer, which is statistically related to independent variables, contains no neurons. On the other hand, all the neurons in the hidden (which is responsible for the major mathematical process) and the output layers (which delivers the dependent variables) take the outputs of their preceding layer as their inputs. Figure 2 shows an example of an ANN architecture with one hidden layer.

For a designed network architecture, defining the initial weights and updating them would be the next step. This step, which is also known as the learning or training algorithm, is in essence the process of minimizing the network error. The training procedure starts by calculating the error with the initial weights and continues with adjusting the interconnecting weights until a maximum iteration level or an acceptable error level is achieved. The network performance can be evaluated, e.g., by the Mean Squared Error (MSE) between the desired and the predicted values of the output:

(2) $$E=\frac{1}{2N}\overset{N}{\underset{i=1}{\sum }}{\left[t_{i}-a_{i}\right]}^{2}$$

where N stands for the number of training sample points, t is the desired value and a is the predicted value for the output of the i-th sample point. As the network error is calculated, the weights and biases are updated through back propagation to reduce the error value. This process is repeated until the error becomes minimized. In each iteration, an adjusted weight is calculated based on:

(3) $$W_{ji}^{\left(n\right)}\left(k\right)=W_{ji}^{\left(n\right)}\left(k-1\right)-\alpha \left(\frac{\partial E}{\partial W_{ji}^{\left(n\right)}}\right)$$

where $W_{ji}^{\left(n\right)}\left(k\right)$ is an updated weight of the n-th layer in the k-th iteration (also known as epoch). $\frac{\partial E}{\partial W_{ji}^{\left(n\right)}}$ is the partial derivative of the error. In this equation, α is the learning rate, which is less than 1. Calculation of the derivative part of the equation is normally achieved by the chain rule [27]. In order to develop a robust neural network, datasets are divided into three subsets—training, validation and testing—and the performance of the model is assessed through each of these subsets. The training dataset is employed to update the weights and bias terms, and in order to prevent overfitting of the network and test set, the validation set is employed [28].

### 2.4. Particle Swarm Optimization (PSO)

Particle Swarm Optimization is known as a powerful numerical algorithm to optimize complex functions by finding the best solution in a space of feasible solutions. This technique, which has been inspired by the social behavior of animals, was first introduced by Eberhart and Kennedy [29]. A simple interpretation of PSO is the behavior of a group of birds who are seeking food. None of the birds know where the single piece of food is located. However, they know their distance from the other birds. Therefore, the simplest and fastest way to achieve the food is to follow the closest bird to that food. In essence, it combines local search methods (through self-experience) with global search methods (through neighboring experience). Here, PSO was chosen merely as an example of global search methods (next to other heuristics methods such as genetics algorithm), owing to its simple and intuitive mathematical rules in finding the optimum solution in high-dimensional spaces.

In PSO, each candidate solution is called a “particle”, which is part of a community known as a “swarm”. PSO solves the optimization problem by moving the particles in a space of all feasible solutions, also known as the search space. Each particle has a memory to keep its best experience, and the cooperation of particles helps them find the best global solution in the search space.

**Figure 3:**
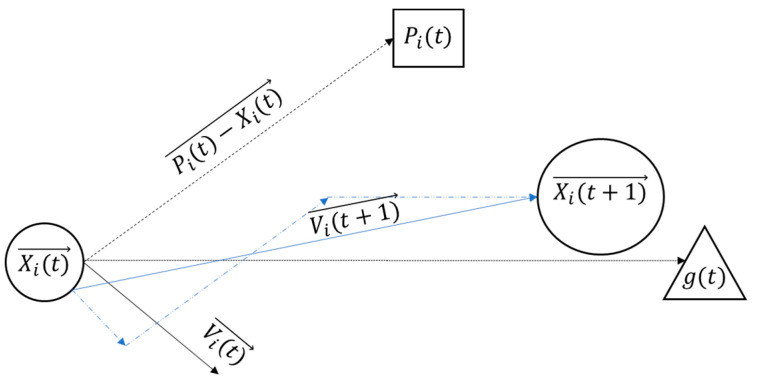
Movement of each particle toward its best position in particle swarm optimization.

As shown in Figure 3, the position of particle i at time step *t*, denoted by $\vec{X_{i}\left(t\right)}$, and its velocity, denoted by $\vec{V_{i}\left(t\right)}$, are the key properties to define a particle. The previous experience of each particle, $\vec{X_{i}\left(t\right)}$, its previous movement $\vec{V_{i}\left(t\right)}$, and the best experience of the whole swarm, g(t), guide each particle moving towards its next position by $\vec{X_{i}\left(t+1\right)}$, which is probably a better experience. This process continues until the swarm meets its best experience (denoted by $P_{i}\left(t\right)$).

In PSO, each particle obeys two rules to update its position and velocity vectors:

(4) $$\vec{X_{i}\left(t+1\right)}=\vec{X_{i}\left(t\right)}+\vec{V_{i}\left(t+1\right)}$$

(5) $$\vec{V_{i}\left(t+1\right)}=wV_{i}\left(t\right)+C_{1}\left(P_{i}\left(t\right)-X_{i}\left(t\right)\right)+C_{2}\left(g\left(t\right)-X_{i}\left(t\right)\right)$$

where w is the inertia coefficient and $C_{1}$ and $C_{2}$ are acceleration coefficients.

The j-th component of new position and speed vectors can be calculated as follows:

(6) $$X_{ij}\left(t+1\right)=X_{ij}\left(t\right)+V_{ij}\left(t+1\right)$$

(7) $$V_{ij}\left(t+1\right)=wV_{ij}\left(t\right)+r_{1}C_{1}\left(P_{ij}\left(t\right)-X_{ij}\left(t\right)\right)+r_{2}C_{2}\left(g_{j}\left(t\right)-X_{ij}\left(t\right)\right)$$

where $V_{ij}\left(t+1\right)$ is the j-th component of velocity of particle i at time step (t+1). In Equation (7), the first component ($wV_{ij}\left(t\right)$) is known as inertia term. The second component is called the cognitive component, and the third term is the social component; $r_{1}$ and $r_{2}$ are the uniformly distributed numbers in the range of 0 and 1. $P_{ij}\left(t\right)$ is the j-th component of position that gives the best value ever experienced by particle *i* and $g_{j}\left(t\right)$ is that experienced by all particles in the swarm.

## 3. Results and Discussion

**Figure 4:**
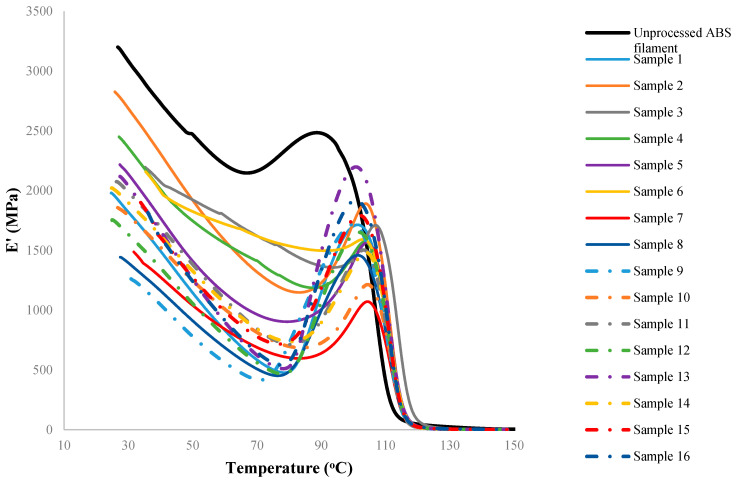
The variation of the storage modulus versus temperature for the test specimens. The overshoot close to glass transition temperature often occurs as the stresses trapped in the part during processing are relieved with an increase in temperature and rearrangement of polymer chains. Note that the sample numbers in the legend correspond to the setups in Table 3, and do not refer to repeats.

**Figure 5:**
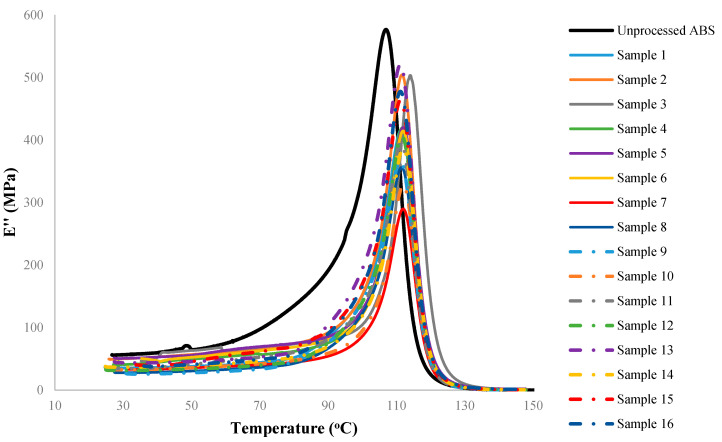
The variation of the loss modulus versus temperature for the test specimens.

**Figure 6:**
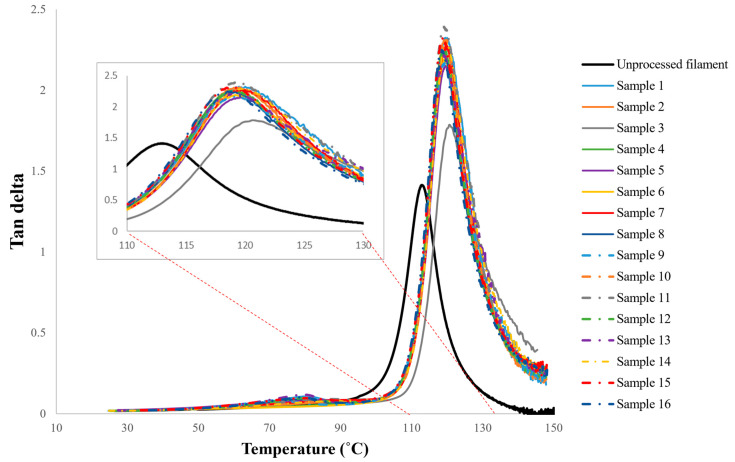
The variation of tan delta versus temperature for the test specimens, with the more detailed view of the variation in the range of 110 °C to 130 °C.

Although there are several process parameters controlling the properties of FDM fabricated parts, this study is focused on four selected factors including the nozzle temperature, the layer height, the raster orientation and the deposition speed. Based on the initial trial and error experiments to ensure that the printed parts have minimum acceptable quality with no visible defects, four levels were assigned to each process parameter. The selected factors and their corresponding levels are presented in Table 2. For this case, the total (full-factorial) number of experiments would be $4^{4}$ or 256. However, assuming that ‘the factor interactions are negligible’, the Taguchi approach [30,31] was implemented to reduce the number of costly DMA experimental runs by means of using orthogonal arrays. Taguchi orthogonal arrays are often shown by “*L_n_*(*x^y^*)”, where *n* stands for the total number of the experiments, *x* represents the levels, and *y* is the number of the controlling factors. Here, an *L*_16_(4^4^) design was used (Table 3, where the levels 1–4 are representing the physical values in Table 2). Variations of the dynamic moduli and tanδ over working temperatures were measured for 16 designed samples of Table 3, and the results are presented in Figure 4, Figure 5 and Figure 6.

According to the observed trends, when comparing the processed (3D printed) and unprocessed samples (as-received filaments), it can be concluded that all the processed samples generally follow a similar trend. However, the comparison between the group of the processed samples and unprocessed samples clearly shows that the FDM has reduced the magnitude of both storage and loss moduli regardless of the combination of process parameters used. The extent of the decrease, however, is highly correlated to the temperature at which the property has been measured in DMA (equivalent to the working temperature of the print in use). As an instance, according to Figure 4, an average reduction of 40% is observed on the storage modulus when pooling all processed samples. This reduction ranges from 15% to 62%, corresponding to samples 2 and 9, respectively. At a specific working temperature, e.g., 100 °C, there is an average reduction of 25% in the storage modulus due to the FDM process. Similarly, the same trends can be seen while measuring the loss modulus of 3D printed ABS samples. As shown in Figure 5, regardless of assigned values to process parameters, the FDM process decreases the loss modulus of fabricated parts compared to the unprocessed sample. For instance, at 40 °C the loss modulus has decreased 11–56% as a result of different FDM process conditions. The reduction at 40 °C (as a nominal working temperature example) has an average value of 33.5%. Nevertheless, the reduction increases drastically and reaches the average value of 60.7% at a working temperature of 100 °C. Regardless of the selected process parameters, on average it is confirmatory to notice that FDM fabricated samples heated up to, e.g., 100 °C show less viscous behavior (represented by the loss modulus) compared to samples tested at 40 °C. Although the FDM process seems to unavoidably decrease the storage and loss moduli of the printed parts, by selecting a suitable (optimized) set of process parameters this reduction may be minimized.

Generally, it is common to identify the glass transition temperature ($T_{g}$) of thermoplastics by measuring the peak of temperature-tanδ curve. Similarly, here Figure 6 was used along with its zoomed view, to find this transitional point of mechanical properties from glassy to rubbery behavior. The results are summarized in Table 4.

**Figure 7:**
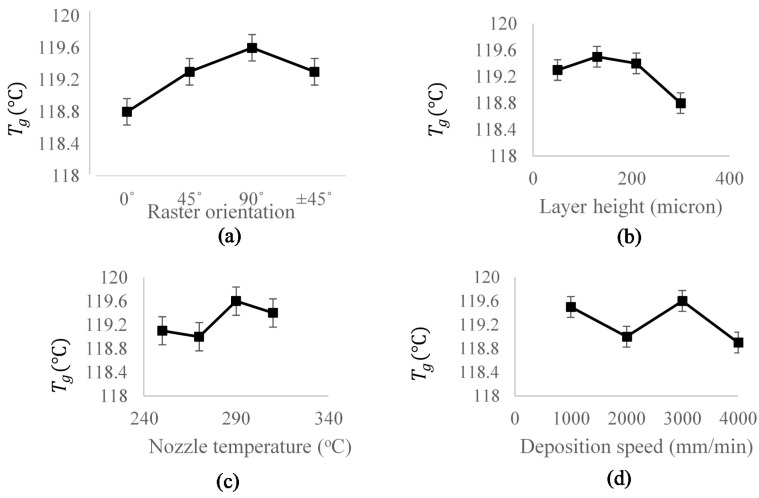
The variation of glass transition temperature of FDM processed ABS as a function of: (**a**) Raster orientation; (**b**) Layer height; (**c**) Nozzle temperature; (**d**) Deposition speed. Note that the presence of air gap (or inversely an overlap) between the printed roads would make a major difference in ensuing macro-level properties and non-linearities observed in the response above, and other mechanical properties as also reported by [19]. The curved shown are average values from all the performed 16 tests.

From the results in Table 4, it can be concluded that the FDM-processed parts have a higher glass transition temperature in comparison to unprocessed ABS; albeit the difference in the values among processed samples themselves is small. In other words, regardless of the combination of the assigned process parameters, FDM processed parts stay longer in the glassy region in comparison to unprocessed material. For instance, according to Table 3, the glass transition temperature has raised from 112.8 °C for unprocessed ABS filament to 120.5 °C under sample 3 (i.e., a 6% increase). Figure 7 shows the effect of each process parameter on the glass transition temperature of the FDM printed parts. In order to statistically rank the effect of process parameters on $T_{g}$, Lenth’s method [32,33] was employed. This method is known as a powerful statistical tool to analyze costly experiments with single replicate factorial designs.

Assuming a design with m effects, considering both factors and interactions, denoted by $c_{1},\;c_{2},\;\ldots ,\;c_{m}$, Lenth’s method performs the effect analysis using a numerical value called the Pseudo Standard Error (PSE). For a $2^{k}$ design, m is equal to $2^{k}-1.$

(8) $$PSE=1.5*median(\left|c_{j}\right|:\left|c_{j}\right|<2.5S_{0}\;$$

where:

(9) $$S_{0}=1.5median\left(\left|c_{j}\right|\right)$$

According to the method, when there is no sufficient information on repeats of a test, PSE is a reasonable measure to estimate the Standard Error [33]. The margin of Error (ME) is the final factor used to compare factor effects:

(10) $$ME=t_{\frac{\alpha }{2},d}PSE$$

where $t_{\frac{\alpha }{2},d}$ is the *t*-distribution with the significance level of α and the degree of freedom of $d=\frac{m}{3}$. Given a specific factor, if the absolute value of the effect is greater than ME , that factor is considered effective (statistically significant). Table 5 represents the mean value of $T_{g}$ under each studied factor, based on Figure 7. The Delta parameter is the difference between the maximum and minimum value of each data column and was considered as the factor effect. Lenth’s parameter (ME threshold) has been calculated via Equations (8)–(10). Comparing the Delta values with each corresponding threshold, it can be concluded that the raster orientation is ranked above all other factors, followed by the feeding rate and layer height, and finally the nozzle temperature, to control glass transition temperature in FDM of ABS parts.

**Figure 8:**
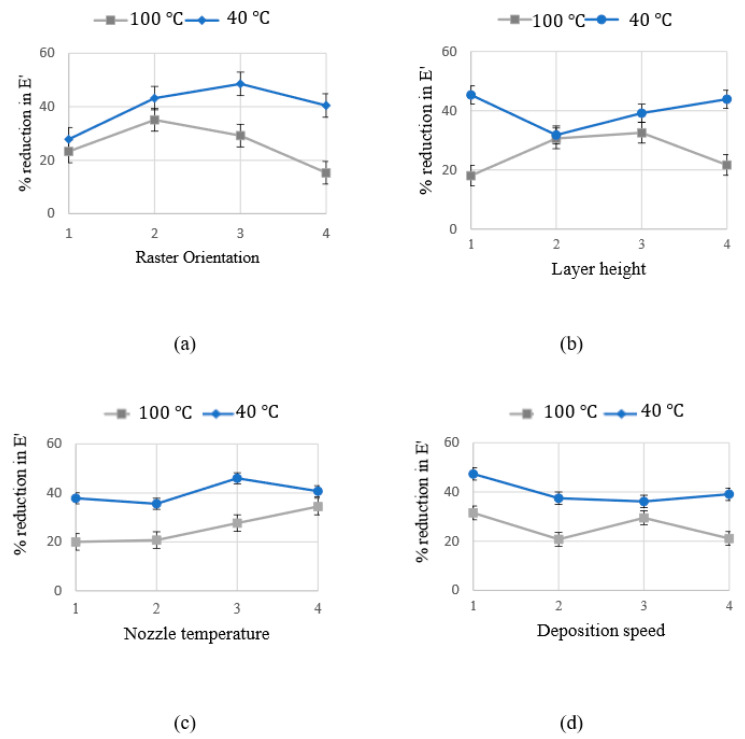
Percentage of reduction in storage modulus as a function of FDM process parameters: (**a**) Raster orientation; (**b**) Layer height; (**c**) Nozzle temperature; (**d**) Deposition speed. The blue and the grey lines represent the average reduction in the storage modulus at 40 °C and 100 °C respectively.

**Figure 9:**
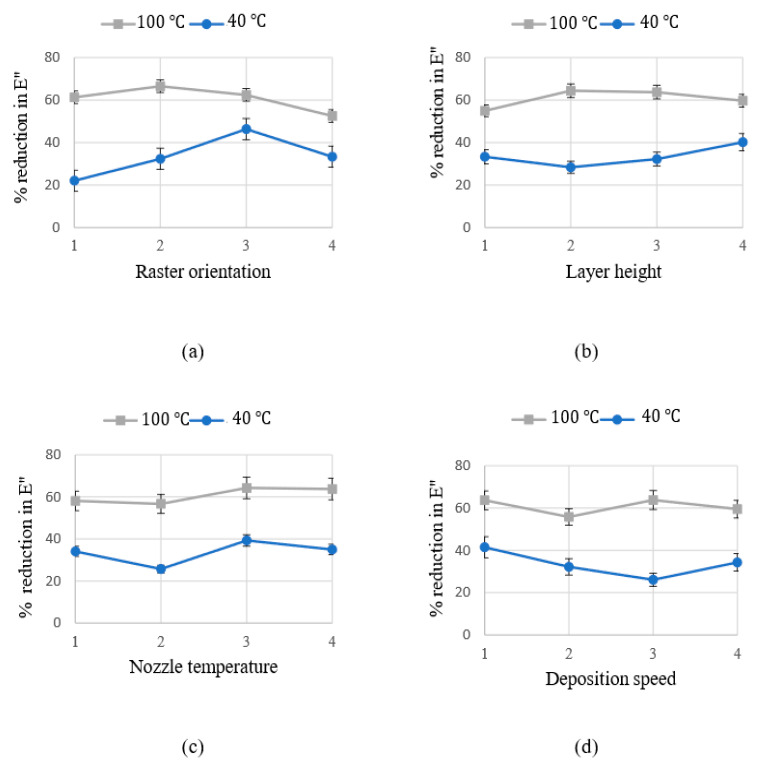
Percentage of reduction in loss modulus as a function of FDM process parameters: (**a**) Raster orientation; (**b**) Layer height; (**c**) Nozzle temperature; (**d**) Deposition speed. The blue and the grey lines represent the average reduction in the loss modulus at 40 °C and 100 °C respectively.

Figure 8 and Figure 9 exemplify the relationship between process parameters and the reductions in dynamic mechanical moduli at two specific working temperatures, including 40 °C and 100 °C. As seen in these figures, the relationship between response modulus and process parameters is sizable, nonlinear and highly dependent on the working temperature.

Owing to the complex nature of the FDM process, the presence of combinative effects of the parameters necessitates the use of advanced modeling techniques to predict the material behavior. As addressed in Section 2.3, the Artificial Neural Network (ANN) is one of the known examples of powerful black-box modeling techniques to approximate such complex non-linear relationships between input and output parameters. In order to collect an adequate size of data to train the ANN model, the moduli values were taken at the working temperature steps of 5 °C from Figure 4 and Figure 5, under each process condition. For each storage and loss modulus response, a separate multi-layer perceptron neural network architecture, including input layer, hidden layer and output layer was designed. After performing a series of training algorithms and testing various ANN architectures, the optimum model was selected for each modulus. Namely, to approximate the storage modulus, a 5-9-1 architecture was employed (five input neurons, nine hidden neurons and one output neuron), while a 5-7-1 architecture was used to simulate the loss modulus.

Both designed networks were trained using 60% of randomly selected data points via Levenberg- Marquardt algorithm. Then, the testing validation for each network was performed using a 20–20% portion of the remaining data. However, it must be mentioned that all data points, except one experimental run (number 9), were used to build the storage modulus and loss modulus networks. The latter experimental run was selected randomly not to be used in training, testing and validation steps. Instead, the data points corresponding to the response curves of test 9 were used to evaluate the robustness of the final developed model for each modulus.

**Figure 10:**
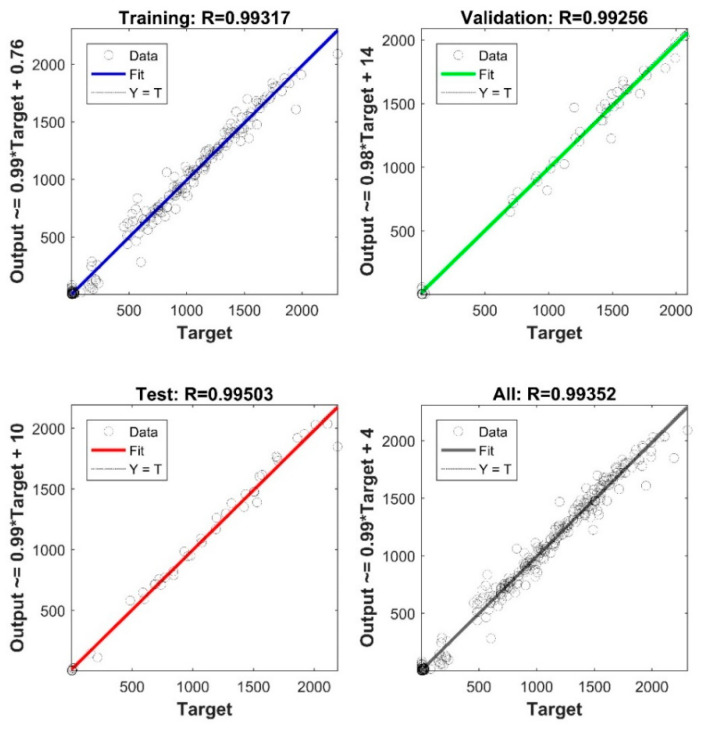
The performance of the developed 5-9-1 neural network to approximate the storage modulus of the 3D prints.

**Figure 11:**
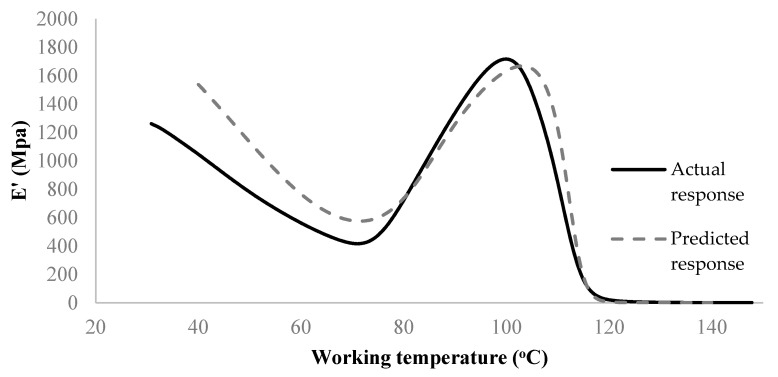
Comparison of the actual and predicted (virtual DMA test of) storage modulus, *E*′, under run #9.

The storage modulus ANN showed an acceptable performance represented by means of the coefficient of correlation (R). The R-values corresponding to training, validation, and testing, respectively, equaled 0.99317, 0-99256, and 0.99503. Figure 10 illustrates the network performance in detail. This designed network was then employed to predict the storage modulus under the fully unseen run #9. Figure 11 depicts the actual values versus the simulated values under this setup.

**Figure 12:**
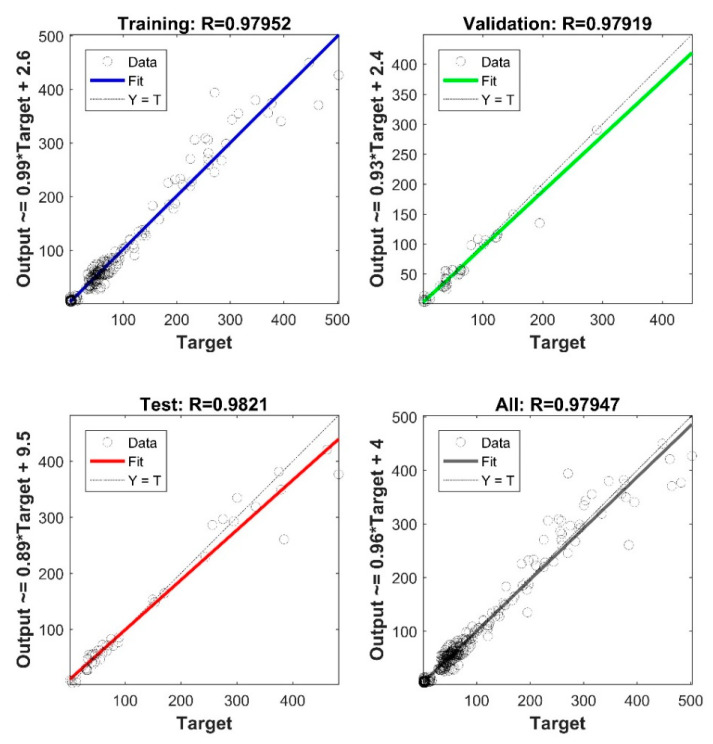
The performance of the developed 5-7-1 neural network to approximate the loss modulus in training, validation and testing.

**Figure 13:**
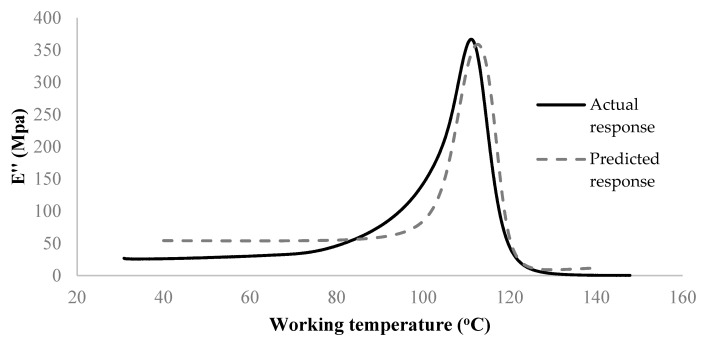
Comparison of the actual and predicted (virtual DMA test of) loss modulus, *E*″, under run #9.

As shown in Figure 11, the developed network is usable to avoid actual experimentation and predict the storage modulus of the untested sample. The deviation between the experiment and model in the early stage of the DMA test can be linked to the large differences seen in the original data (Figure 4) in the same range when comparing different process conditions. Accordingly, as more DMA tests become available for training (here only 16 samples were used), the performance of ANN would have also been improved. Despite limited data, the overall validation R-score of the present model (i.e., considering all samples and all regions of response) is still >99% (Figure 10). Similarly, a 5-7-1 network architecture was selected to predict the loss modulus of FDM fabricated ABS samples. The network performance is illustrated in Figure 12. The final verification of the network performance was completed by predicting set-up condition #9 (Figure 13).

**Figure 14:**
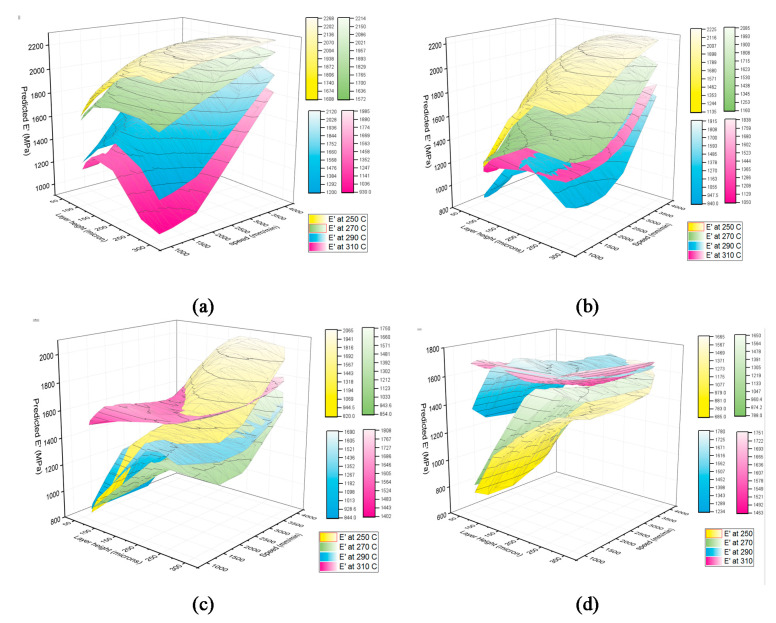
Simulated response of the storage modulus numeral network, at a working temperature of 40 °C and fixed nozzle temperatures (shown by different colors); (**a**) at 0° raster orientation, (**b**) at 90° raster orientation, (**c**) at 45° raster orientation, (**d**) at ±45° raster orientation.

Figure 14 represents an example of a simulated modulus as a function of process parameters, using the above AI model, at a given working temperature of 40 °C. According to Figure 14a, during printing of samples with $0^{{}^{\circ}}$ raster orientation, increasing the nozzle temperature decreases the general trend of storage modulus response. However, as shown in Figure 14b, increasing the nozzle temperature, first decreases and then increases the storage modulus. Moreover, both mentioned figures show that at higher values of the layer height, the storage modulus is higher. This trend is also visible for prints at $45^{{}^{\circ}}$ raster orientation, as depicted in Figure 14c. The effect of nozzle temperature on the general trend of storage modulus based on the layer height and deposition speed at raster orientation of $45^{{}^{\circ}}$ is highly nonlinear where the contours are crossing each other (indicating a high level of interaction of process parameters). Finally, as illustrated in Figure 14d, at the raster orientation of $\pm 45^{{}^{\circ}}$, regardless of the value assigned to the nozzle temperature, the higher the layer height, the higher the storage modulus. Factor interaction effects can also be clearly observed in the $\pm 45^{{}^{\circ}}$ raster orientation case.

Noticing the above complex relationship between the process parameters and their interactions on the ensuing dynamic moduli of the prints, which in turn also vary at each given target working temperature, performing a nonlinear optimization of the process is deemed vital for FDM designers to ensure maximized performance of printed parts during use. Accordingly, the optimum set of process parameters would be the one at which the fabricated part shows the highest value of storage or loss moduli, i.e., as close as possible to the parent (unprocessed) material. Here, using the developed ANNs, such an optimum set of process parameters at each working temperature condition was obtained using the Particle Swarm Optimization (PSO) technique. As per Table 2, the layer height was allowed to change from 50 μm to 300 μm, the nozzle temperature from 250 °C to 310 °C and the deposition speed from 1000 mm/min to 4000 mm/min, at each level of raster orientation. The working temperature was varied between 40 °C and 140 °C. The PSO was performed in MATLAB (R2016b, MathWorks, Natick, MA, USA) using 100 particles in each swarm. In order to end the iterating process, the maximum number of iterations was set at 1000 and the function tolerance was defined to be 10^−25^. The minimum and maximum inertia weights were chosen to be 0.1 and 1.1, respectively. It should be noted that prior to the optimization process, the data points were normalized to be between 0 and 1.

**Figure 15:**
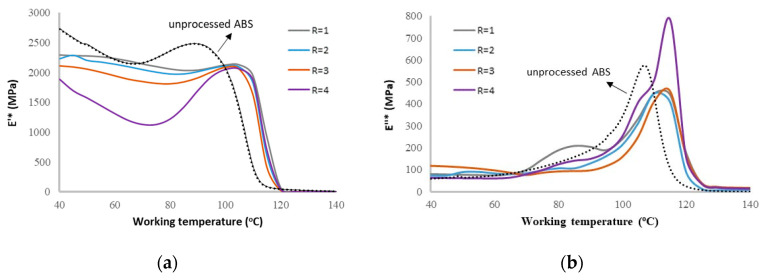
Comparison of the optimized (**a**) storage and (**b**) loss moduli of the prints, as compared to the parent (unprocessed) material, at different working temperatures and raster orientations (R). 4 levels provided for R are corresponding to the levels presented in Table 2.

The obtained optimum values of the process parameters along with the maximum achievable storage modulus are presented in Table A1. Table A2 similarly illustrates the optimization results for the loss modulus. The latter optimum values were reported for each working temperature. Results clearly show that there is no single optimum process condition that can optimize the printed part performance at all working temperature conditions. In practice, such look-up tables may be used by a designer, e.g., to choose the best FDM condition given the target operating temperature (application) of the 3D printed part. To better visualize the efficiency of the optimization, Figure 15 shows the optimized moduli of prints compared to the unprocessed material. When comparing Figure 15a with Figure 4, it can be noticed that the optimized prints exhibit closer behavior to that of the unprocessed material, albeit the storage modulus of the unprocessed material prior to *T_g_* is still relatively higher than the prints. A similar effect of optimization, though to a lesser extent, can be noticed in regard to the loss moduli of the prints (compare Figure 5 and Figure 15b). From Figure 15, it can be induced that the optimum moduli values for raster orientations $0^{{}^{\circ}}$, $90^{{}^{\circ}}$, and $45^{{}^{\circ}}$ are comparable, but at the orientation $\pm 45^{{}^{\circ}}$ a distinct behavior is evidenced, namely providing a much lower storage modulus (pre-glass transition) but a higher storage modulus upon process optimization.

## 4. Conclusions

In this paper, first, the variation of the viscoelastic response of 3D printed ABS samples in a wide range of working temperatures (the room condition to 140 °C) was studied as a function of four FDM process parameters (raster orientation, layer height, nozzle temperature, and deposition speed). The experimental layout was designed via a Taguchi orthogonal array in order to minimize the size of data required for subsequent ANN training. Dependency of the viscoelastic properties of the printed samples on the process control factors was shown via Lenth’s method. Consequently, the optimum parameter values corresponding to each working condition were obtained using the ANN models (for storage and loss moduli), integrated with the PSO algorithm. The optimum values were reported for various working temperatures for both moduli. Results clearly showed that there is no single optimum process condition that can optimize the printed part performance at all working temperature conditions. However, specific conclusions could be drawn from the observations, as follows.

- The FDM process condition could directly affect the maximum allowable working temperature (represented by glass transition temperature) for the 3D printed thermoplastic.
- Based on Lenth’s statistical analysis, among the process parameters, raster orientation was the most effective factor to increase the glass transition temperature of the 3D printed parts. Subsequently, the deposition speed and the layer height were ranked second, followed by the nozzle temperature.
- Distinct trends between viscoelastic responses of unprocessed and processed ABS filaments under various process conditions pointed to the fact that all FDM process conditions significantly (on average 40%) lowered the magnitude of viscoelastic moduli regardless of a specific combination of process parameters, which is also in agreement with the earlier study [5]. This effect is deemed critical for designers to consider for the reliable application of 3D printed parts, especially at high temperatures.
- Although it was shown that there are distinct trends between the behavior of processed and unprocessed ABS samples, the exact change in the moduli was highly dependent on the working temperature, at which the part viscoelastic properties were measured. For instance, at a working temperature of 100 °C, there was an average reduction of 25% in storage modulus when compared to the unprocessed sample. On the other hand, this reduction at a 40 °C working temperature was about 33.5%. The reduction increased drastically and reached as high as 60.7% at high working temperatures >100 °C.
- It was validated that the developed neural network architectures are capable of predicting the entire DMA curve of 3D printed parts, including for samples that were fully unseen to the original model. Using such networks, optimum values of process parameters can be obtained via global search methods such as particle swarm optimization for each given target working temperature (Table A1 and Table A2). The optimized prints indicated a closer behavior to that of the parent material.
- The optimized prints with orientation $\pm 45^{{}^{\circ}}$ showed clearly a distinct behavior compared to the $0^{{}^{\circ}}$, $90^{{}^{\circ}}$, and $45^{{}^{\circ}}$ orientations.

Further study may be worthwhile to test the presented AI modeling approach for other thermoplastics, and possibly for improving the permeance of the predictions by employing and comparing other high-fidelity machine learning methods. Furthermore, the observed variation of dynamic mechanical properties can be further supported with, e.g., a molecular weight analysis of samples.

## Figures and Tables

**Table 1:** Properties of the ABS filaments.

Commercial code	CHIMEI PA-747S
Nominal diameter (mm)	1.75
Purity	>98%
Nominal Young’s modulus (GPa)	2
Relative density—$H_{2}O$ $\left(\frac{g}{{\mathrm{cm}}^{3}}\right)$	1.03–1.10
Decomposition temperature (°C)	>310

**Table 2:** Control factors used in the experimental procedures with their assigned levels.

Control Factors	Level 1	Level 2	Level 3	Level 4
Raster orientation	0° 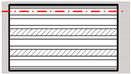	90° 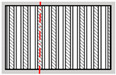	45° 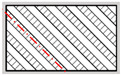	±45° 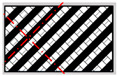
Layer height (μm)	50	130	210	300
Temperature (°C)	250	270	290	310
Feeding rate (mm/min)	1000	2000	3000	4000

**Table 3:** Orthogonal array used to design the experimental layout with respect to configuration levels given in Table 2.

Sample #	Raster Orientation	Layer Height (μm)	Nozzle Temperature (°C)	Deposition Speed (mm/min)
1	1	1	1	1
2	1	2	2	2
3	1	3	3	3
4	1	4	4	4
5	2	1	2	3
6	2	2	1	4
7	2	3	4	1
8	2	4	3	2
9	3	1	3	4
10	3	2	4	3
11	3	3	1	2
12	3	4	2	1
13	4	1	4	2
14	4	2	3	1
15	4	3	2	4
16	4	4	1	3

**Table 4:** Values of glass transition temperature measured by DMA.

Sample	$T_{g}$ (°C)	Sample	$T_{g}$ (°C)
1	119.466	9	119.268
2	119.363	10	119.682
3	120.578	11	119.131
4	119.156	12	118.986
5	119.608	13	118.918
6	119.178	14	119.771
7	119.711	15	117.984
8	118.667	16	118.514
		Unprocessed ABS filament	112.854

**Table 5:** Lenth’s method of factor analysis for glass transition temperature; the values shown correspond to the average of response under each corresponding level of the process factors. The physical values of the levels were given in Table 2.

Level	Raster Orientation	Layer Height	Temperature	Deposition Speed
**1**	118.8	119.3	119.1	119.5
**2**	119.6	119.5	119	119
**3**	119.3	119.4	119.6	119.6
**4**	119.3	118.8	119.4	118.9
Delta	0.8	0.7	0.6	0.7
ME threshold	0.515	0.515	0.515	0.515

## Data Availability

Data is contained within the article.

## Appendix A. Optimization Results Raw Data

**Table A1:** The optimum values (look-up table) of the process parameters using PSO on E′ at each raster orientation level and under each working temperature.

R	LH* (μm)	NT* (°C)	DS* $\left(\frac{mm}{min}\right)$	WT (°C)	E’* (MPa)	R	LH* (μm)	NT* (°C)	DS* $\left(\frac{mm}{min}\right)$	WT (°C)	E’* (MPa)
1	300	310	1275	40	2292	2	270	250	3374	40	2227
1	300	310	1281	45	2282	2	279	250	3357	45	2285
1	300	310	1295	50	2277	2	286	250	3338	50	2199
1	50	310	1464	55	2263	2	288	250	3320	55	2172
1	50	310	1527	60	2234	2	292	250	3314	60	2136
1	50	310	1567	65	2193	2	299	250	3318	65	2093
1	50	310	1575	70	2141	2	300	250	3332	70	2046
1	50	310	1548	75	2099	2	300	250	3344	75	2003
1	50	310	1484	80	2058	2	300	250	3341	80	1972
1	50	310	1378	85	2033	2	300	250	3314	85	1973
1	76	310	1211	90	2039	2	300	250	3268	90	2011
1	87	310	1000	95	2080	2	300	250	3229	95	2066
1	94	310	1000	100	2125	2	300	252	3263	100	2105
1	102	310	1000	105	2128	2	300	253	3081	105	2088
1	118	310	1000	110	1945	2	300	253	2841	110	1817
1	50	268	3956	115	922	2	300	251	2564	115	607
1	50	269	3941	120	71	2	300	252	2601	120	37
1	175	310	1237	125	6	2	300	250	2894	125	4
1	300	297	1000	130	2	2	300	250	3215	130	2
1	300	296	1000	135	2	2	300	250	3452	135	2
1	300	298	1048	140	2	2	300	250	3538	140	2
3	300	310	4000	40	2111	4	300	310	2023	40	1887
3	235	250	4000	45	2092	4	177	296	1000	45	1692
3	242	250	4000	50	2056	4	194	297	1000	50	1573
3	251	250	4000	55	2004	4	207	297	1000	55	1443
3	265	250	4000	60	1944	4	220	298	1000	60	1312
3	287	250	4000	65	1886	4	239	297	1000	65	1196
3	300	250	4000	70	1848	4	300	292	1000	70	1131
3	300	250	4000	75	1817	4	300	292	1000	75	1130
3	300	250	4000	80	1810	4	300	293	1000	80	1222
3	300	250	4000	85	1842	4	300	294	1000	85	1423
3	300	250	4000	90	1910	4	167	310	1000	90	1696
3	300	250	3774	95	2008	4	167	310	1000	95	1931
3	300	250	3639	100	2086	4	173	310	1000	100	2051
3	300	250	3577	105	2056	4	190	310	1000	105	2060
3	300	250	3548	110	1620	4	229	310	1000	110	1873
3	300	310	1000	115	414	4	223	310	1000	115	704
3	300	310	1000	120	22	4	300	310	1000	120	46
3	300	250	3861	125	3	4	300	310	1000	125	4
3	300	250	4000	130	2	4	300	250	4000	130	2
3	300	250	4000	135	2	4	300	250	4000	135	2
3	300	250	4000	140	2	4	162	250	4000	140	2

* LH*, NT*, DS* stand for the optimum Layer height, Nozzle temperature, Deposition speed and given Raster orientation R and Working temperature WT. E’* is the corresponding optimum storage modulus.

**Table A2:** The optimum values (look-up table) of the process parameters using PSO on E″ at each raster orientation level and under each working temperature.

R	LH* (μm)	NT* (°C)	DS* $\left(\frac{mm}{min}\right)$	WT (°C)	E″* (MPa)	R	LH* (μm)	NT* (°C)	DS* $\left(\frac{mm}{min}\right)$	WT (°C)	E″* (MPa)
1	50	310	4000	40	79.8	2	54	277	4000	40	74.1
1	50	310	4000	45	78.3	2	50	276	4000	45	73.9
1	50	310	4000	50	76.7	2	300	250	1000	50	90.5
1	50	310	4000	55	75.3	2	300	250	1000	55	91.2
1	50	310	4000	60	73.4	2	278	250	1000	60	84.4
1	50	273	1000	65	80	2	300	310	4000	65	80.2
1	300	250	1000	70	99.3	2	300	310	4000	70	85.6
1	300	250	1000	75	146.7	2	50	250	4000	75	98.2
1	300	250	1000	80	188.8	2	50	250	4000	80	107.6
1	75	250	1000	85	208.7	2	50	250	4000	85	107.4
1	300	250	1000	90	204.5	2	300	296	4000	90	129.6
1	300	250	1000	95	189.6	2	300	297	4000	95	163.1
1	50	250	3548	100	239.6	2	300	298	4000	100	213.8
1	98	250	3967	105	334	2	300	298	4000	105	313.5
1	110	250	4000	110	448.5	2	50	279	4000	110	448.9
1	115	254	4000	115	442.2	2	50	282	4000	115	407.6
1	284	278	4000	120	176.4	2	300	310	4000	120	89.4
1	300	283	4000	125	34	2	300	310	3860	125	13.6
1	300	285	4000	130	17.2	2	300	271	1000	130	7.7
1	186	280	4000	135	14.7	2	50	310	1586	135	8.2
1	148	281	4000	140	14.4	2	50	310	1895	140	9.9
3	50	250	4000	40	117.7	4	300	310	1000	40	63.7
3	50	250	4000	45	114.9	4	300	310	1000	45	62.6
3	50	250	4000	50	111	4	133	250	1000	50	61.9
3	50	250	4000	55	105.2	4	148	250	1000	55	61.6
3	50	250	4000	60	97	4	170	250	1000	60	60.8
3	50	250	1000	65	86.2	4	170	310	4000	65	64.7
3	50	250	1000	70	76	4	150	310	4000	70	79.6
3	50	250	1000	75	86.1	4	155	250	4000	75	99.3
3	50	250	1000	80	92.4	4	140	250	4000	80	125
3	50	250	1000	85	93.9	4	134	250	4000	85	141.8
3	300	250	3364	90	96.9	4	131	250	4000	90	149.9
3	258	310	1000	95	116.7	4	50	310	1000	95	181.2
3	255	310	1000	100	158.2	4	50	310	1000	100	252.5
3	251	310	1000	105	251.9	4	50	250	1000	105	408.6
3	102	250	4000	110	412.5	4	50	250	1000	110	508.5
3	67	250	4000	115	458.6	4	300	250	2278.8	115	783.6
3	50	250	4000	120	164.1	4	50	250	1000	120	155.1
3	50	250	4000	125	37.3	4	50	250	1000	125	34.2
3	50	250	4000	130	21.6	4	50	250	1000	130	19.5
3	50	250	4000	135	18.2	4	50	250	1000	135	15.8
3	50	250	3911	140	16.7	4	50	250	1000	140	14.2

* LH*, NT*, DS* stand for the optimum Layer height, Nozzle temperature, Deposition speed and given Raster orientation R and Working temperature WT. E″* is the corresponding optimum loss modulus.
